# Variability in intracellular localization of D‐amino acid oxidase in choroid plexus epithelial cells

**DOI:** 10.1111/febs.70459

**Published:** 2026-02-17

**Authors:** Koji Ono, Yuji Shishido, Toshiyuki Yamagishi, Kiyoshi Fukui

**Affiliations:** ^1^ School of Medical Technology, Faculty of Health and Medical Care, Saitama Medical University Saitama Japan; ^2^ Institute of Photonics and Human Health Frontier (IPHF) Tokushima University Tokushima Japan; ^3^ Institute for Enzyme Research (KOSOKEN) Tokushima University Tokushima Japan

**Keywords:** autophagosome, choroid plexus epithelial cells, D‐amino acid oxidase, endosome, exocytosis, lysosome, peroxisome, trans‐Golgi network

## Abstract

D‐amino acid oxidase (DAO; also abbreviated as DAAO) in the brain is primarily expressed in glial cells of the cerebellum and brainstem. In glial cells, DAO metabolizes D‐serine, which acts as a co‐agonist and regulator of glutamate receptors. We previously reported that DAO is also expressed in epithelial cells of the choroid plexus (CP); however, the subcellular localization and functional significance of DAO in the CP remain unclear. In this study, we performed histological analyses to investigate the subcellular localization of DAO in the CP. We first confirmed DAO expression in the mouse CP. Immunostaining revealed vesicle‐like DAO signals in the cell bodies of choroid plexus epithelial cells (CPECs). Super‐resolution microscopy was then used to evaluate colocalization with intracellular organelle markers. DAO signals partially colocalized with ATP‐binding cassette subfamily D member 3 (PMP70) and peroxisomal targeting signal 1 receptor (PEX5). In contrast, they colocalized with Golgin subfamily A member 2 (GM130), trans‐Golgi network integral membrane protein 2 (TGN46), adaptor protein complex AP‐2 (AP‐2), early endosome antigen 1 (EEA1), Ras‐related proteins Rab5a, Rab11, CD63 antigen (CD63), tumor susceptibility gene 101 protein (TSG101), lysosome‐associated membrane glycoprotein 1 (LAMP‐1), LAMP‐2, beclin‐1 and microtubule‐associated protein 1 light chain 3 (LC3). These findings suggest that, in the CP, DAO is transported from the Golgi apparatus to endosomes and subsequently distributed to multiple vesicular compartments. The presence of DAO in peroxisomes, autophagosomes, lysosomes, and exosomes indicated diverse intracellular localization within CPECs. This distribution may enable efficient metabolism of blood‐derived D‐serine in CPECs.

## Graphical abstract

D‐amino acid oxidase (DAO) in choroid plexus epithelial cells (CPECs) shows vesicle‐like localization by histological and super‐resolution analyses. DAO colocalizes with peroxisomal, Golgi, endosomal, lysosomal, autophagosomal, and exosomal markers, indicating diverse subcellular distribution. This suggests DAO is transported within CPECs to metabolize blood‐derived D‐serine, contributing to cerebrospinal fluid D‐serine regulation and highlighting its potential physiological role in the choroid plexus.

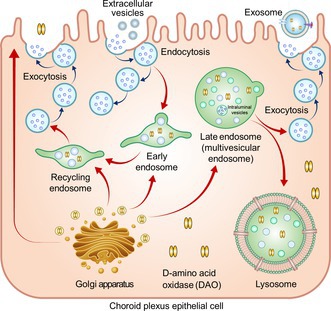

**Abbreviations**
AP‐2: transcription factor AP‐2
CD63: CD63 antigen
CP: choroid plexus
CPECs: choroid plexus epithelial cells
CSF: cerebrospinal fluid
DAB: 3,3′‐diaminobenzidine
DAO: D‐amino acid oxidase
EEA1: early endosome antigen 1
GM130: Golgin subfamily A member 2
ILVs: intraluminal vesicles
LAMP‐1: lysosome‐associated membrane glycoprotein 1
LAMP‐2: lysosome‐associated membrane glycoprotein 2
LC3: microtubule‐associated protein 1 light chain 3
MVEs: multivesicular endosomes/late‐endosomes
PEX5: peroxisomal targeting signal 1 receptor
PMP70: ATP‐binding cassette subfamily D member 3
PTS‐1: peroxisomal targeting signal‐1
Rab11: Ras‐related protein Rab11
Rab5a: Ras‐related protein Rab5a
TGN46: trans‐Golgi network integral membrane protein 2
TSG101: tumor susceptibility gene 101 protein

## Introduction

D‐amino acid oxidase (DAO, also abbreviated as DAAO; EC1.4.3.3) is a flavoenzyme that catalyzes the oxidation of D‐amino acids into their corresponding imino acids and hydrogen peroxide (H_2_O_2_) [1]. The imino acids are then nonenzymatically hydrolyzed to α‐keto acids and ammonia [2]. In mammals, high levels of DAO expression are observed in the kidney, liver, cerebellum, brainstem, and spinal cord [3]. In this study, we adopt the abbreviation DAO [4, 5, 6], which is commonly used in neuroscience literature, and explicitly distinguish it from diamine oxidase (EC1.4.3.22).

Regarding the intracellular localization of DAO, no reports were published from its initial discovery in 1935 until the 1960s. In 1965, cellular fractionation of rat liver revealed that DAO activity was concentrated in a specific peroxisome‐like cellular fraction [7]. Electron microscopic observations of DAO began in the 1980s. Using DAO activity staining and immunostaining with anti‐DAO antibodies, studies in rat liver and kidney confirmed localization within peroxisomes, as DAO colocalized with catalase. Furthermore, immunostaining in rat liver confirmed that DAO is enriched in a core‐like subcompartment of peroxisomes [8, 9]. Previous studies have refined our understanding of DAO's cellular and subcellular localization in the human brain, especially within the choroid plexus tissue (CP). Immunohistochemical and *in situ* hybridization analyses of postmortem human brain tissue revealed DAO immunoreactivity not only in glial cells of the rhombencephalon but also in choroid plexus epithelial cells (CPECs) [10].

Recent studies tracking the lifespan and degradation pathways of human DAO have shown that human DAO in the peroxisomal fraction is degraded via the lysosomal/endosomal pathway, whereas cytoplasmic human DAO is degraded by the proteasome [11]. This indicates that DAO is not ‘permanently anchored to peroxisomes’ but is functionally linked to the endosome–lysosome system. Furthermore, DAO carrying ALS‐associated mutations R199W and E121K is associated with LC3‐positive autophagosome formation and increased autophagic flux, suggesting that DAO aggregation and quality control are mediated by the autophagy system [12, 13]. Given DAO's peroxisomal targeting signal type‐1 (PTS‐1) at its C terminus (Ser‐His‐Leu) and previous demonstrations of DAO localization to peroxisomes in multiple mammalian tissues [8, 9, 14, 15, 16], these findings raise the question of whether DAO in CPECs is localized exclusively to peroxisomes or whether it also traffics through, or is active in, other intracellular compartments (e.g., Golgi, endosomes, autophagosomes).

Most recently, large‐scale proteomic studies that include CP (or the CSF proteome reflecting CP secretion) have demonstrated that components of protein degradation pathways, endocytosis, and lysosomal function are dysregulated in the CP in Alzheimer's disease, suggesting active metabolic and enzymatic functions in the CPECs and associated subcellular organelles [17]. Moreover, the possibility that DAO expression in the CP contributes to the modulation of D‐serine (or other D‐amino acids) levels in cerebrospinal fluid (CSF), and that this regulation may be altered in disease (e.g., schizophrenia, Alzheimer's disease), remains of high interest.

The presence of DAOs in CPECs has been reported, but direct evidence regarding which organelles are involved and to what extent within CPECs remains at the estimation stage. In this study, we focused on examining DAO protein expression, particularly its intracellular localization and transport system, in mouse CPECs using high‐resolution immunofluorescence, confocal imaging, and colocalization analysis with organelle markers. Our objective was to determine whether the site of D‐amino acid metabolism within the CP can be inferred by exploring (i) whether DAO in the mouse CP is confined to peroxisomes or also localizes to nonperoxisomal organelles, and (ii) whether a transport mechanism exists that contributes to diversity in the intracellular localization of DAO.

## Results

### Antibody specificity and DAO expression in the mouse CP

**Fig. 1:**
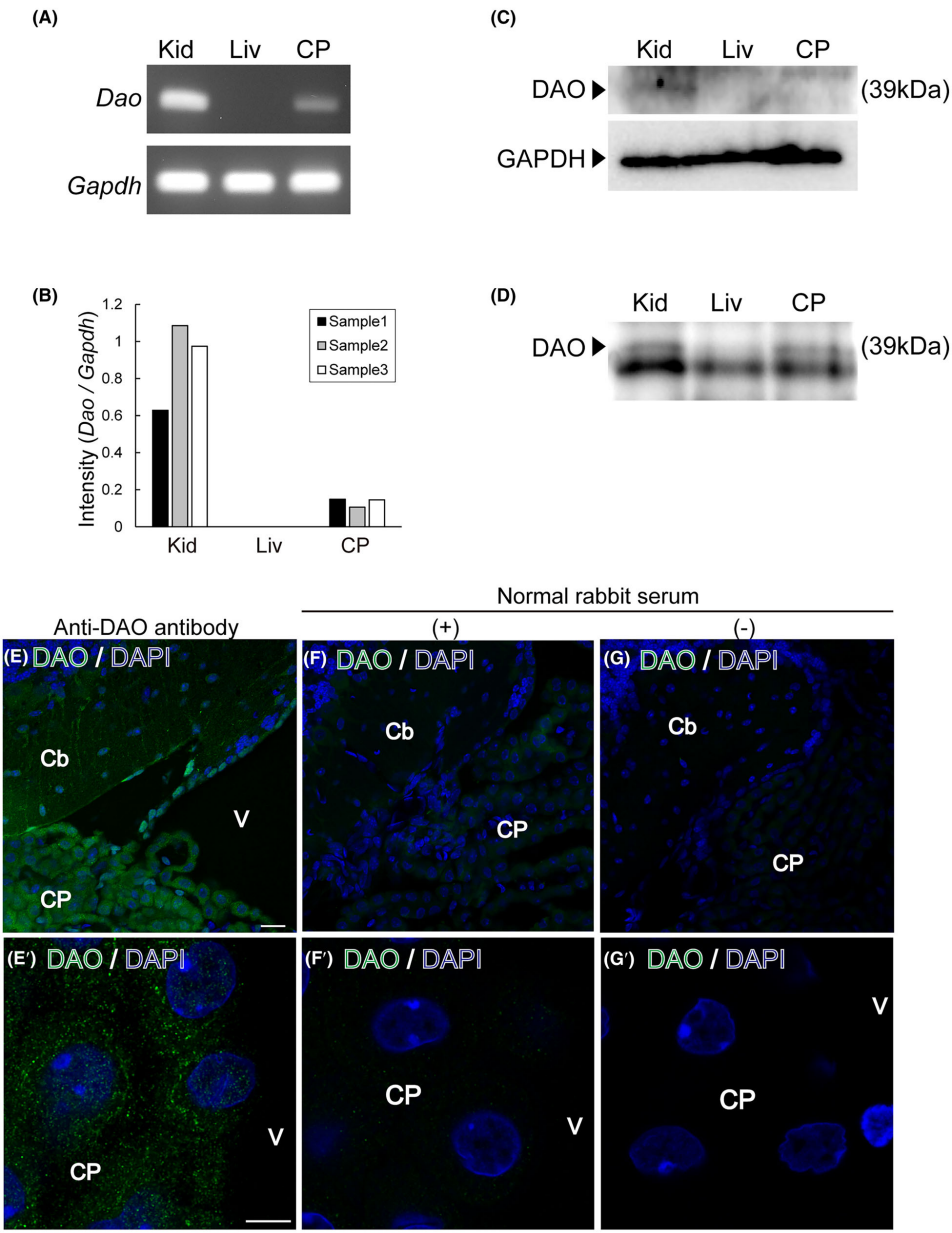
Antibody specificity of the anti‐D‐amino acid oxidase antibody. (A, B) D‐amino acid oxidase (DAO) gene expression in mouse tissues (kidney, liver, and choroid plexus) was confirmed by RT‐PCR. (A) Results of agarose gel electrophoresis are shown. The upper lane shows amplified gene fragments of the DAO gene, while the lower lane shows amplified fragments of the GAPDH gene as a control (*n* = 3). (B) Results from independent experiments using three mice are shown. The signal intensity of the bands obtained in (A) was analyzed. (C) DAO protein expression in mouse tissues was confirmed by western blotting. The upper lane shows the DAO protein, while the lower lane shows the GAPDH protein as a control (*n* = 3). (D) Immunoprecipitates using anti‐DAO antibodies were analyzed by western blotting with anti‐DAO antibodies (*n* = 4). (E–G) Immunostaining using anti‐DAO antibodies revealed DAO‐positive signals in the CP (*n* = 3). DAO signals were detected as green fluorescence, while cell nuclei were detected as blue fluorescence. The immunostaining conditions were: (E) primary antibody with anti‐DAO antibody, (F) primary antibody with normal rabbit serum, and (G) primary antibody with blocking buffer only. (E′–G′) Enlarged views of the choroid plexus portion shown in (E–G). CP, choroid plexus; Cb, cerebellum; Kid, kidney; Liv, liver; V, ventricle. Scale bars indicate 20 μm (E–G) and 5 μm (E′–G′).

To verify whether the anti‐DAO antibody previously used in human and rat choroid plexus [10] also recognizes DAO derived from mouse tissues, RT‐PCR, western blotting, and immunostaining were performed. First, RT‐PCR was conducted to identify tissues expressing or not expressing mouse DAO (Fig. 1A,B). The results showed that, in mice, DAO was expressed in the kidney but not in the liver. Furthermore, DAO expression was detected in the choroid plexus, albeit at lower levels than in the kidney. Next, western blotting was performed to verify whether the anti‐DAO antibody used in this study recognizes mouse DAO protein (Fig. 1C,D). A DAO‐positive band was detected at the expected molecular weight, with expression patterns consistent with those of the DAO gene.

**Fig. 2:**
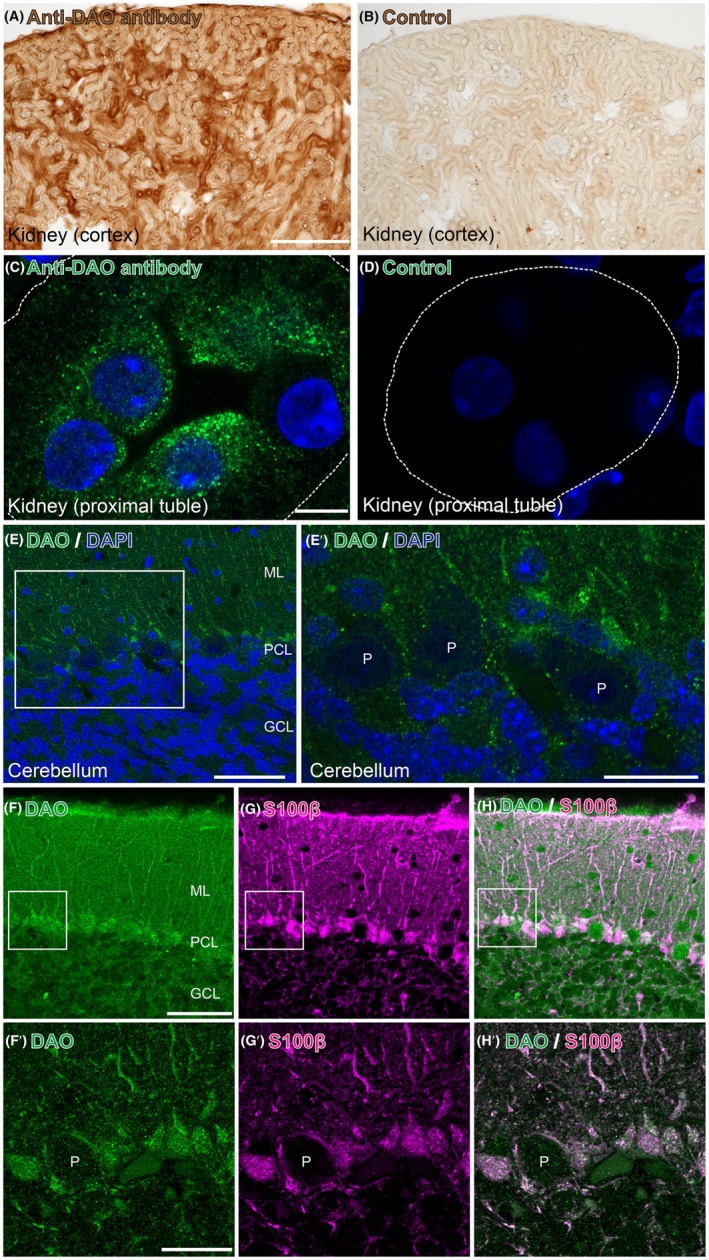
Immunostaining of kidney and cerebellum using anti‐D‐amino acid oxidase antibody. Immunostaining with anti‐D‐amino acid oxidase (DAO) antibody in kidney (*n* = 3) and cerebellum (*n* = 3) tissue is shown. (A and B) Low magnification view of the renal cortex. DAB staining was performed, with DAO‐positive signals indicated in brown. (C and D) High magnification view of a horizontal section of the proximal tubule in the kidney. Fluorescent staining was performed, with DAO‐positive signals indicated in green and nuclei in blue. The dotted line indicates the basement membrane. (B and D) The findings show the control (no antibody) at low magnification and high magnification, respectively. (E, E′) DAO expression in three cerebellar layers was confirmed via confocal laser scanning microscopy. Immunopositive signals for DAO (green) were observed in the Purkinje cell layer and the molecular layer. (F–H) Colocalization of DAO and S100β in the cerebellum was confirmed. Immunofluorescent signals of DAO (green) were mainly observed with glial marker S100β (magenta). (F′–H′) Enlarged images indicate that DAO‐expressing cells in the cerebellum were not Purkinje cells but the surrounding glial cells expressing S100β. GCL, granular cell layer; ML, molecular layer; P, Purkinje cell; PCL, Purkinje cell layer. Scale bars indicate 50 μm (A and B), 5 μm (C and D), 50 μm (E–H), and 20 μm (E′–H′).

Finally, we confirmed whether the anti‐DAO antibody could be used for immunohistochemical staining of the target CP (Fig. 1E–G,E′‐G′). The results showed that the anti‐DAO antibody detected signals likely representing DAO in the cerebellum and choroid plexus (DAO: green, nuclei: blue) (Fig. 1E,E′). As antibody negative controls, immunostaining was performed using normal rabbit serum instead of the primary antibody (Fig. 1F,F′) and blocking solution without serum (Fig. 1G,G′). In these control experiments, no DAO‐positive signals were detected. Additionally, immunohistochemical staining was performed on kidney and cerebellum tissue, where DAO expression is known to occur (Fig. 2). DAO was expressed in the proximal tubules of the kidney cortex (Fig. 2A,C), whereas no positive signal was observed in the control (Fig. 2B,D). DAO was expressed in glial cells in the cerebellar cortex (Fig. 2E,E′). DAO localization was confirmed using anti‐S100β antibody, a marker for glial cells (Fig. 2F–H,F′‐H′). In the cerebellum, DAO was observed in glial cells in the Purkinje cell layer of the cortex. Therefore, the results confirm that the anti‐DAO antibody used in this study is suitable for detecting mouse DAO in tissues.

### Distribution of DAO in the mouse brain

**Fig. 3:**
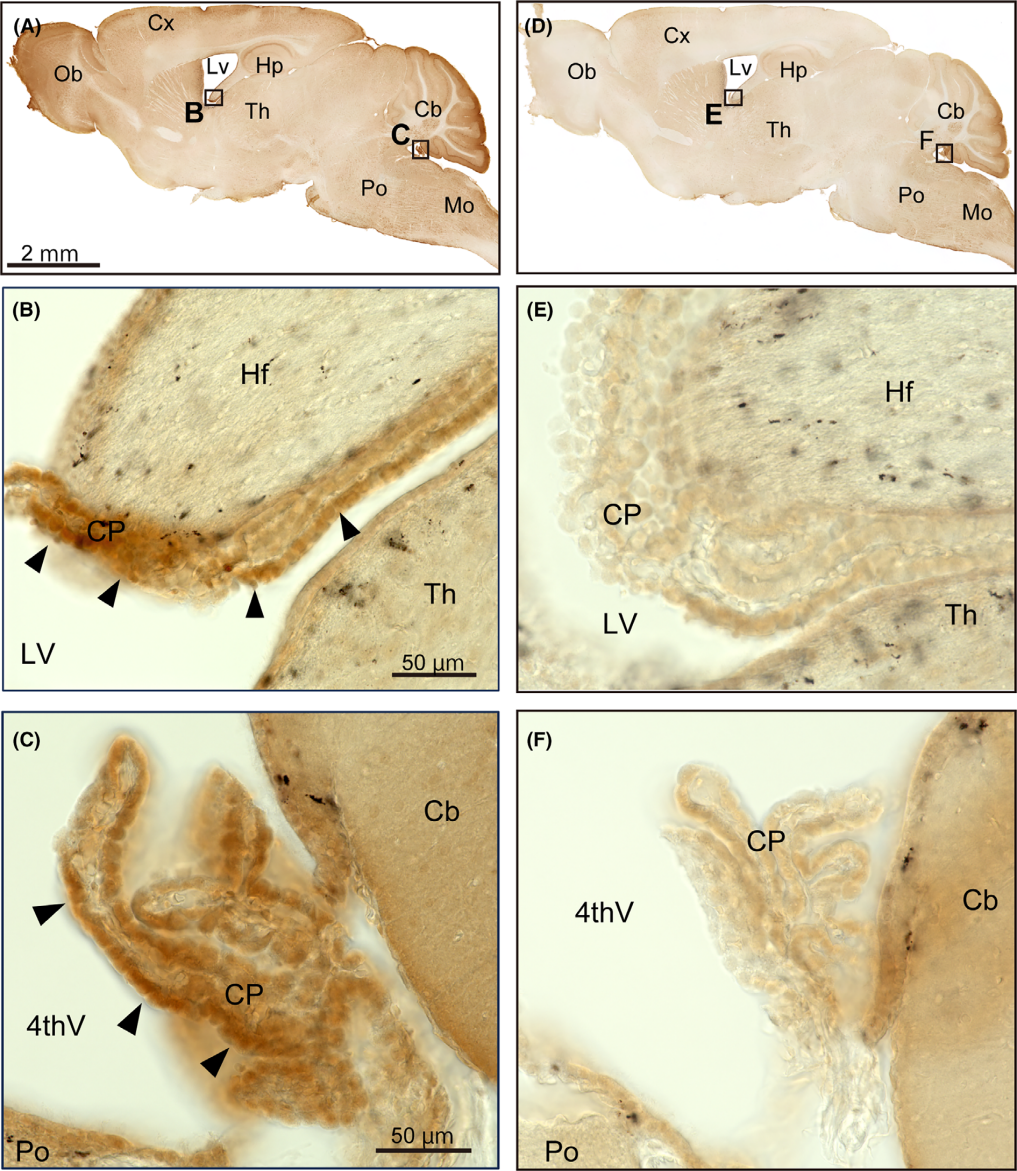
Immunohistochemical imaging of D‐amino acid oxidase in the mouse brain. (A) Immunohistochemical signals of D‐amino acid oxidase (DAO) (brown) were observed in the pons, medulla oblongata, cerebellum, and choroid plexus (*n* = 3). DAO‐immunopositive signals (indicated by arrow heads) were observed in the choroid plexus of the lateral ventricle (B) and the fourth ventricle (C). (D–F) The images shown represent controls for A–C (antibody‐free controls). 4thV, fourth ventricle; Cb, cerebellum; CP, choroid plexus; Cx, cerebral cortex; Hp, hippocampus; Lv, lateral ventricle; Mo, medulla oblongata; Ob, olfactory bulb; Po, pons; Th, thalamus. Scale bars indicate 2 mm (A and D) or 50 μm (B, C and E, F).

To determine whether DAO expression is present in the CP of the mouse ventricle, as in the rat CP, we performed immunostaining on sagittal sections of the mouse brain using anti‐DAO antibodies (Fig. 3A–F). As a result, DAO‐positive signals were detected in the lateral ventricular CP (Fig. 3B) and the fourth ventricular CP (Fig. 3C) of the mouse brain, in addition to the pons, medulla oblongata, and cerebellum, consistent with findings in the rat brain (Fig. 3A).However, no positive signal was observed in the control (Fig. 3D–F).

**Fig. 4:**
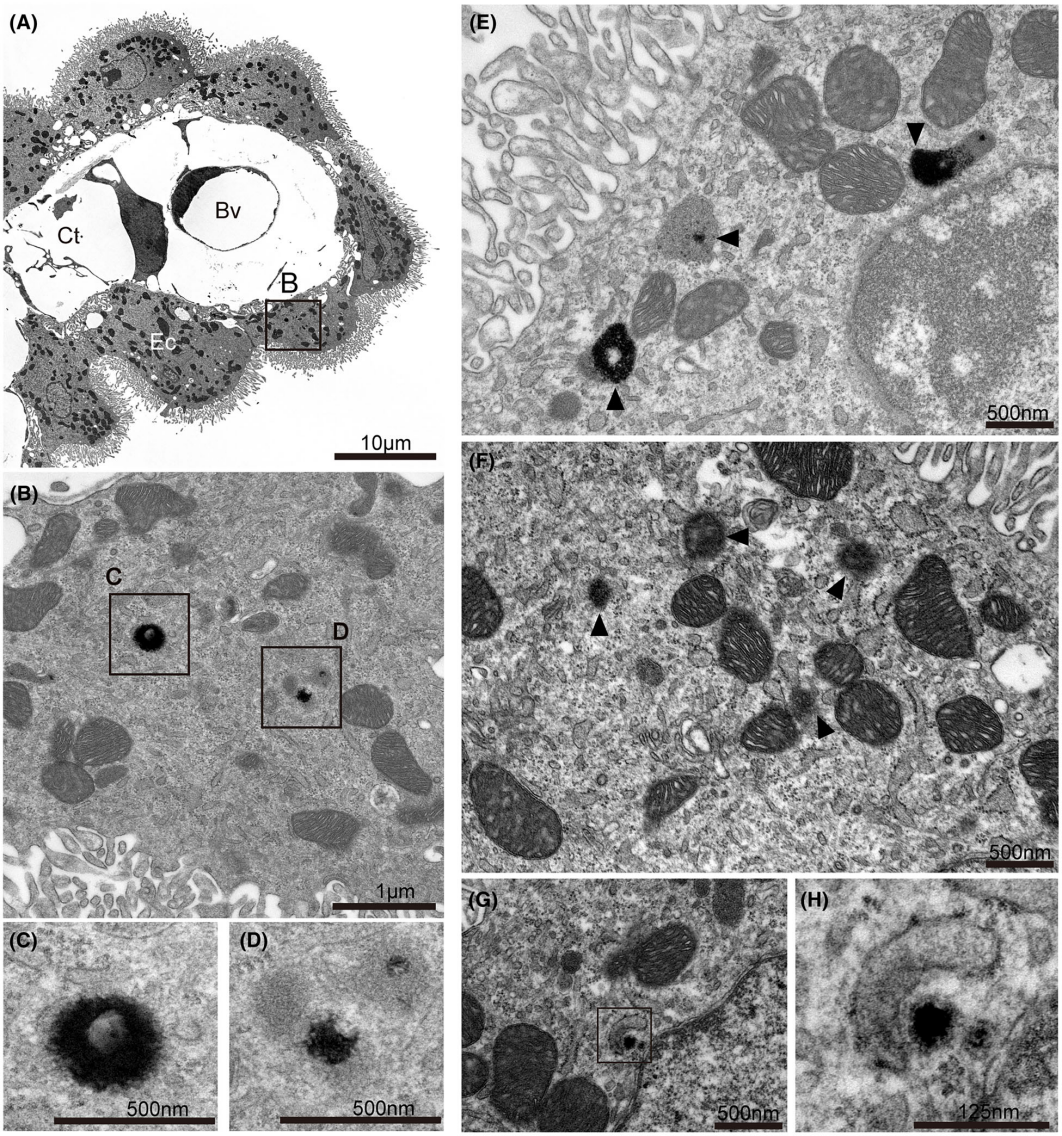
Immunoelectron microscopic images of D‐amino acid oxidase in the mouse choroid plexus. (A–H) Immunostaining with the anti‐D‐amino acid oxidase (DAO) antibody was observed via transmission electron microscopy (*n* = 3). (A) The tissue structure of the choroid observed by immunoelectron microscopy is shown. (B) This image shows a magnified view of the entire choroid plexus epithelium. The dense black signal within the square box indicates DAO‐positive staining. (C, D) A highly magnified image of the square within the choroid plexus epithelial cells (B) is shown. (E) DAO‐immunopositive signals in choroid plexus cells were observed in shapes other than round. (F) Weak DAO‐positive signals were also detected. (G, H) The cell membrane was seen enveloping the DAO‐positive signal, suggesting phagocytosis. Arrow heads indicate DAO‐immunopositive signals in E and F. Bv, blood vessel; Ct, connective tissue; Ec, epithelial cell. Scale bars indicate 10 μm (A), 1 μm (B), 500 nm (C–G), and 125 nm (H).

To confirm the distribution of DAO in CPECs, we performed immunostaining combined with electron microscopy. First, we observed tissue stained with 3,3′‐diaminobenzidine (DAB) under an optical microscope to examine the immunostaining results and confirmed DAO expression in CPECs via electron microscopy (Fig. 4A–H). Our findings indicated that the DAO‐positive signals appeared in a vesicle‐like shape (Fig. 4A). DAO‐positive signals showed a strong trend in vesicular‐like structures (Fig. 4B–D), while their size and shape were heterogeneous (Fig. 4E). Weak DAO‐positive signals with indistinct cell membranes were also observed (Fig. 4F). In some cases, DAO‐positive signals were observed being engulfed by cell membranes, suggesting phagocytosis (Fig. 4G,H). However, electron microscopy did not identify a consistent cell organelle in which the DAO‐positive signals were localized.

### Localization of DAO in cell organelles

To identify the cell organelles where DAO is localized, we performed multiple fluorescence immunostaining and observed the results using a super‐resolution confocal laser microscope (LSM900) equipped with Airyscan 2. Conventional confocal microscopes are limited by optical diffraction to approximately 200 nm in the XY direction and 500–700 nm in the Z direction. In contrast, the LSM900 confocal microscope equipped with Airyscan2 can improve the resolution to approximately 120 nm in the XY direction and approximately 350 nm in the Z direction. This improvement is achieved by efficiently collecting photons with a detector array and reconstructing the point spread function during postprocessing [18, 19]. Therefore, unlike other super‐resolution microscopes (such as STED or SIM), this system does not require specialized conditions and can be used with sample preparation similar to that of confocal microscopy. For these reasons, we determined that this microscope was suitable for observing the organelles in this study and selected it for our analysis.

**Fig. 5:**
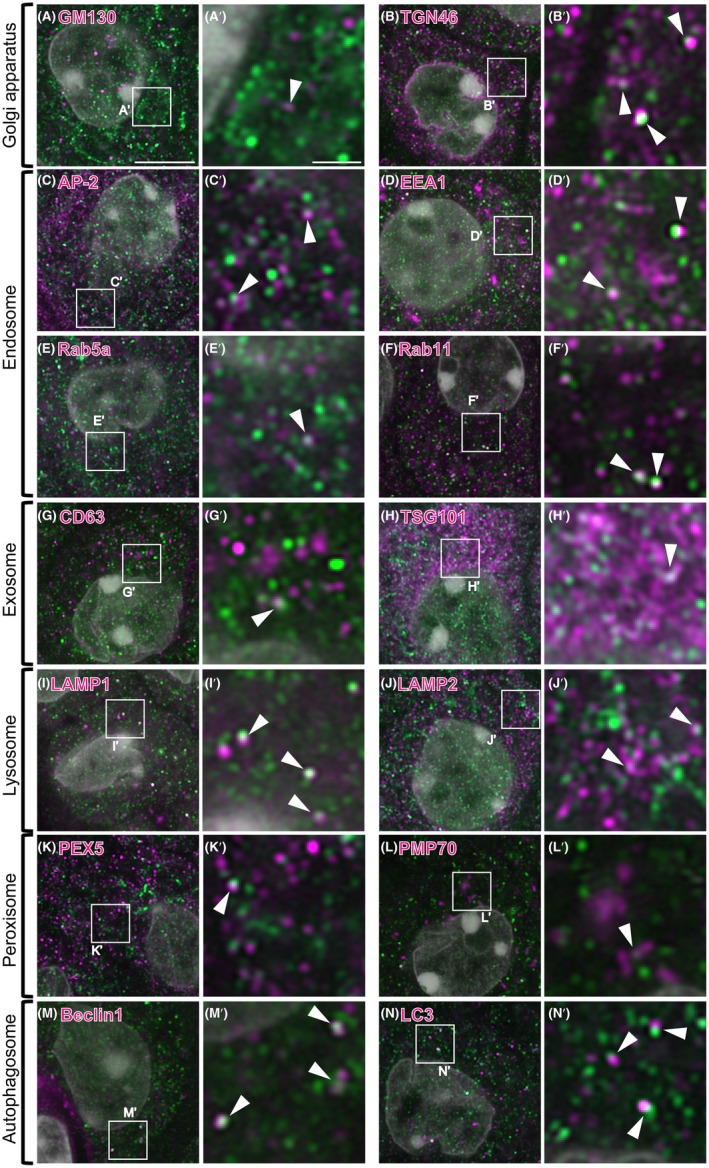
Intracellular localization of D‐amino acid oxidase using organelle‐specific markers. Representative immunofluorescence images show the localization of D‐amino acid oxidase (DAO, green) corresponding to various organelles and vesicle markers (magenta). Nuclei were counterstained with DAPI (blue). Immunohistochemistry was performed for each organelle and vesicle marker in independent experiments (*n* = 4). Since it was not possible to analyze all markers in the same individual mouse, a total of 11 mice were used for immunohistochemical analysis. (A) DAO with cis‐Golgi matrix protein GM130; (B) DAO with trans‐Golgi network marker TGN46; (C) DAO with adaptor protein complex 2 (AP‐2), involved in clathrin‐dependent endocytosis; (D) DAO with early endosome antigen 1 (EEA1), an early endosome marker; (E) DAO with Rab5a, a small GTPase regulating early endosome dynamics; (F) DAO with Rab11, a marker of recycling endosomes; (G) DAO with CD63, a tetraspanin abundant in multivesicular bodies (MVBs) and exosomes; (H) DAO with tumor susceptibility gene 101 protein (TSG101), a component of the ESCRT‐I complex; (I) DAO with LAMP1 and (J) with LAMP2 (both lysosomal membrane glycoproteins); (K) colocalization of DAO with PEX5 (cytoplasmic receptor for peroxisome targeting signal 1 (PTS1) containing protein); (L) DAO with PMP70 (peroxisome membrane protein involved in metabolite transport); (M) DAO with Beclin‐1, an autophagy‐related protein involved in autophagosome formation; and (N) DAO with LC3, a widely used autophagosome marker. (A′–N′) Super‐resolution images correspond to the boxed regions in A–N. Arrowheads indicate colocalized areas (yellow). Scale bars indicate 5 μm (A–N) and 1 μm (A′–N′).

Immunostaining with the DAO antibody showed punctate DAO signals in the cell bodies of CPECs (Fig. 4). Comparing the immunostaining results with electron microscopy findings, DAO was also observed in vesicles with both strong and weak signals in the immunostaining results (Figs 4 and 5).

We examined whether DAO utilizes the Golgi apparatus transport pathway. The results showed that DAO exhibited slight colocalization with GM130 (Golgin subfamily A member 2), a marker for the cis‐Golgi network (Fig. 5A,A′). In contrast, DAO showed relatively extensive colocalization with TGN46 (trans‐Golgi network integral membrane protein 2), a marker for the trans‐Golgi network (Fig. 5B,B′). These results suggest that DAO may be transported to various organelles via the trans‐Golgi network in CPECs.

Since DAO colocalizes with the trans‐Golgi network, we next examined the presence of DAO in various endosomes, which constitute the transport pathways within the trans‐Golgi network. Analysis was performed using specific antibodies against AP‐2 (transcription factor AP‐2) (Fig. 5C,C′), the early endosome marker EEA1 (early endosome antigen 1) (Fig. 5D,D′), Rab5a (Ras‐related protein Rab5a) (Fig. 5E,E′), the recycling endosome marker Rab11 (Ras‐related protein Rab11) (Fig. 5F,F′), and the multivesicular endosome and exosome markers CD63 (CD63 antigen) (Fig. 5G,G′) and TSG101 (tumor susceptibility gene 101 protein) (Fig. 5H,H′). Observations revealed that DAO colocalization with endosomes was relatively high in early endosomes and recycling endosomes, moderate in exosomes, and tended to be low in MVBs/exosomes.

Furthermore, we examined the localization of DAO relative to lysosomes. The results revealed that DAO signals colocalized with LAMP1 (lysosome‐associated membrane glycoprotein 1) (Fig. 5I,I′). Similarly, LAMP2 (lysosome‐associated membrane glycoprotein 2) signals (Fig. 5J,J′), another lysosomal marker, also colocalized with DAO. Regarding the localization of peroxisomes and DAO, for which colocalization has been previously reported, moderate colocalization with PEX5 (peroxisomal targeting signal 1 receptor) (Fig. 5K,K′) was confirmed, whereas only slight colocalization with PMP70 (ATP‐binding cassette subfamily D member 3) (Fig. 5L,L′) was observed. Furthermore, we examined the localization of DAO and autophagosomes (vesicles belonging to the same metabolic degradation pathway as peroxisomes). The results showed that the DAO signals moderately colocalized with Beclin‐1 (Fig. 5M,M′) and only slightly with the LC3 (Microtubule‐associated protein 1 light chain 3) signal (Fig. 5N,N′).

**Fig. 6:**
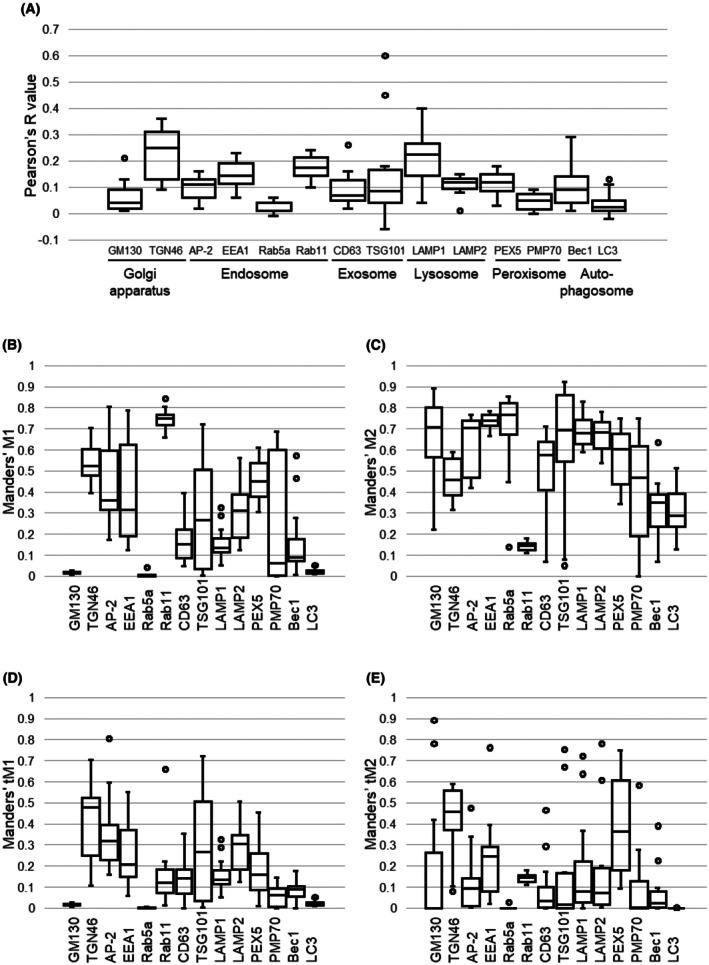
Quantitative colocalization analysis. Pearson's correlation coefficient (*R* value) was calculated to evaluate the degree of colocalization between D‐amino acid oxidase (DAO) and each organelle‐specific marker. Each data point represents cells extracted from the immunohistochemical images in Fig. 5: GM130 (*n* = 17), TGN46 (*n* = 11), AP‐2 (*n* = 11), EEA1 (*n* = 16), Rab5a (*n* = 14), Rab11 (*n* = 12), CD63 (*n* = 16), TSG101 (*n* = 12), LAMP1 (*n* = 20), LAMP2 (*n* = 14), PEX5 (*n* = 18), PMP70 (*n* = 13), beclin1 (*n* = 15), LC3 (*n* = 18)) are presented in a box plot showing the distribution of *R* values obtained by independent calculations. The median line within each box indicates the median value; box boundaries represent the interquartile range (25th percentile), and whiskers indicate the minimum and maximum values within 1.5 times the interquartile range. Individual points outside the whiskers correspond to outliers. Higher *R* values (Pearson's correlation coefficients) (A), M1 values, (the fraction of organelle marker signal overlapping with DAO) (B), M2 values, (the fraction of DAO signal overlapping with organelle markers) (C), tM1 and tM2 (Thresholded M1 and M2 calculated using Costes' automatic thresholding method to minimize background contributions) (D and E) reflect strong spatial colocalization between DAO and each marker. AP‐2, transcription factor AP‐2; CD63, CD63 antigen; EEA1, early endosome antigen 1; GM130, Golgin subfamily A member 2; LAMP1, lysosome‐associated membrane glycoprotein 1; LAMP2, lysosome‐associated membrane glycoprotein 2; LC3, Microtubule‐associated protein 1 light chain 3; PEX5, peroxisomal targeting signal 1 receptor; PMP70, ATP‐binding cassette subfamily D member 3; Rab11, Ras‐related protein Rab11; Rab5a, Ras‐related protein Rab5a; TGN46, trans‐Golgi network integral membrane protein 2; TSG101, tumor susceptibility gene 101 protein.

Quantitative colocalization analysis was performed to assess the subcellular distribution of DAO relative to various organelle markers (Fig. 6 and Table S1). Pearson's correlation coefficients (*R* values) revealed that overall colocalization between DAO and the tested markers was low to moderate, with most values ranging from 0.05 to 0.25. Among these, DAO exhibited relatively higher correlation with the trans‐Golgi network marker TGN46 (0.25 ± 0.10) and the lysosomal marker LAMP1 (0.23 ± 0.11). In contrast, negligible correlations were observed with Rab5a (0.01 ± 0.02) and the autophagosome marker LC3 (0.03 ± 0.04), indicating minimal colocalization with early endosomes or autophagosomes. Manders' coefficients provided further insights into the extent of signal overlap. M1 values, which represent the fraction of organelle marker signal overlapping with DAO, were particularly high for Rab11 (0.75 ± 0.05) and TGN46 (0.53 ± 0.09), suggesting that a substantial portion of these organelle signals colocalized with DAO. However, M1 values for GM130, Rab5a, and LC3 were close to zero, indicating little overlap.

M2 values (Fig. 6 and Table S1), which indicate the fraction of DAO signal overlapping with organelle markers, were generally higher than M1 values across most markers. Notably, strong overlaps were observed with early and recycling endosome markers (EEA1, 0.74 ± 0.03; Rab5a, 0.77 ± 0.20) and with lysosomal markers (LAMP1, 0.68 ± 0.07; LAMP2, 0.68 ± 0.08). These results suggest that a large proportion of DAO resides within endosomal and lysosomal compartments. In contrast, the overlap of DAO with autophagosomal markers (Beclin‐1, 0.35 ± 0.15; LC3, 0.29 ± 0.11) was limited.

Thresholded Manders' coefficients (tM1 and tM2) (Fig. 6 and Table S1), calculated using Costes' automatic thresholding method to minimize background contributions, supported these findings. While TGN46, Rab11, and LAMP2 retained measurable colocalization after thresholding, Rab5a and LC3 values were nearly zero, indicating negligible colocalization. Taken together, these results indicate that DAO shows partial colocalization with the trans‐Golgi network and recycling endosomes, but a stronger and more consistent overlap with early endosomal and lysosomal compartments. DAO also exhibited moderate association with peroxisomal markers (PEX5, PMP70), consistent with its known peroxisomal localization. In contrast, colocalization with autophagosomal markers was minimal, suggesting that DAO is not substantially associated with autophagosome structures under the conditions tested.

## Discussion

In this study, we histologically analyzed the variability of DAO localization in murine CPECs. The results showed that DAO localizes not only to peroxisomes but also to the Golgi apparatus, endosomes, lysosomes, phagosomes, and exosomes. These findings suggest that DAO matures in the Golgi apparatus, is transported via the trans‐Golgi network, and plays a role in each vesicle. In other words, DAO in lysosomes and phagosomes may metabolize D‐amino acids in degradation products, whereas DAO in endosomes and exosomes may be secreted and metabolize D‐amino acids outside the cell.

Previous reports have suggested that DAO is localized to peroxisomes [9, 14]. This is consistent with our electron microscopy observations and the presence of a type 1 peroxisomal targeting migration signal at the C terminus of the DAO amino acid sequence. DAO has also been reported in the peroxisomes of the renal proximal tubular epithelium [9, 14]. To confirm antibody cross‐reactivity, immunolabeling of proximal tubular epithelial cells was performed using the antibodies employed in this study (Fig. 1). The results confirmed the presence of diverse vesicles similar to those observed in the CP (Fig. 2). These findings suggest that DAO may exhibit varying dynamics in different cell types. Future studies are needed to analyze the mechanisms underlying these differences in intracellular dynamics.

We also observed variability in DAO distribution in CEPCs (Figs 2, 5, 6). The results revealed the intracellular pathways that may be involved in DAO transport. DAO is sorted from the Golgi apparatus into early endosomes via the trans‐Golgi network after protein biosynthesis, without undergoing the anticipated transport pathway to peroxisomes [20, 21]. In addition, some vesicles from the trans‐Golgi network are secreted extracellularly via exocytosis [22]. Early endosomes internalize intraluminal vesicles (ILVs) and mature into multivesicular endosomes (MVEs) [21]. Furthermore, MVEs can migrate to the plasma membrane, fuse, and release endosomal ILVs as exosomes [21]. In contrast, MVEs may fuse with lysosomes and autophagosomes to degrade proteins and recycle degradation products as nutrients. Based on our findings regarding DAO localization in these vesicles, two hypotheses can be proposed: (1) DAO is present in various parts of the cell to metabolize its substrate D‐serine; and (2) DAO contained in vesicles of the trans‐Golgi network or in exosomes is released from CPECs into the CSF.

The first hypothesis posits that DAO localization is crucial for D‐serine regulation because DAO must bind to D‐serine to degrade its substrate. Numerous studies indicate that D‐serine plays an essential role in the mouse brain [2, 23, 24]. Within this context, our study predicts that DAO metabolizes metabolized D‐serine as it crosses the blood‐cerebrospinal fluid barrier, which is composed of choroid plexus epithelial cells, stromal cells, and vessel endothelial cells, that is, during its transition from blood to cerebrospinal fluid (CSF). If D‐serine moves rapidly from blood to CSF, blood and CSF D‐serine concentrations should be in equilibrium. However, studies comparing D‐serine concentrations in blood and CSF have reported higher concentrations in blood than in CSF [25, 26]. Considering that CPECs are the site of CSF production, this suggests that the central role of DAO in CPECs is to regulate D‐serine concentrations in CSF.

The second hypothesis is that DAO may function as part of the barrier function present in epithelial tissues. To date, DAO expression has been confirmed in the proximal tubules of the kidney (tubular epithelial cells) [8], the vestibular epithelium of the inner ear (transitional cells) [27], and the small intestine [28, 29]. DAO is thought to be involved in the regulation of D‐amino acids at each of these barrier sites. Furthermore, in the mucosal epithelium of the small intestine, its function suggests that DAO is secreted extracellularly [29]. The results of this study suggest that DAO may also be released extracellularly in CPECs, similar to goblet cells. Immunostaining results for CD63 and TSG101 (Fig. 5G,H,G′,H′) suggest DAO may be secreted via the trans‐Golgi network or the exosome pathway. The function of secreted DAO may be to maintain a constant D‐serine concentration regulated by CPECs, even during circulation within the ventricles. Subsequently, D‐serine reaching the synaptic cleft is further regulated by DAO expressed in astrocytes. We believe that this multistage mechanism of D‐serine concentration control within the brain is appropriate for its strict regulation.

These results support the hypothesis that DAO in CPECs metabolizes D‐amino acids before peripherally derived D‐amino acids enter the CSF. In this study, the localization of DAO was visualized histologically in the choroid plexus of normal mice. The findings of this study are limited by the one‐way analysis based solely on histology. For further investigation, we consider it appropriate to perform dynamic analysis at the cellular level rather than at the tissue level. In other words, we aim to dynamically analyze the subcellular localization of DAO *in vitro* and, based on these results, extend our analysis to animal models of schizophrenia. This approach will enable further elucidation of the pathophysiology of schizophrenia.

## Materials and methods

### Animals

All procedures used in this study were conducted in accordance with the Japan Guidelines for Animal Care and were approved by the Animal Care and Use Committee of Saitama Medical University (approval no. 4083). All efforts were made to minimize the number of animals used in the study. Male C57BL/6J mice (8–10 weeks old, CLEA Japan Inc., Tokyo, Japan) were housed under conventional conditions at 22 ± 2°C with a 12‐h light/dark cycle and *ad libitum* access to food and water. Environmental enrichment was provided. Various surgical procedures were performed following intraperitoneal administration of anesthetics (0.3 mg·kg^−1^ medetomidine hydrochloride (Nippon Zenyaku Kogyo Co., Ltd., Fukushima, Japan), 4 mg·kg^−1^ midazolam (Astellas Pharma Inc., Tokyo, Japan), and 5 mg·kg^−1^ butorphanol tartrate (Meiji Seika Pharma Co., Ltd., Tokyo, Japan)), and efforts were made to minimize animal suffering. Ten mice were used for RT‐PCR (*n* = 3), western blotting (*n* = 3), and immunoprecipitation experiments (*n* = 4). Eleven mice were used for immunohistochemical experiments of DAO in the brain, and three mice were used for immunoelectron microscopy analysis of DAO in CPECs.

### RNA isolation and RT‐PCR

Total RNA was extracted from each tissue (kidney, liver, and CP) using the PureLink RNA Mini Kit (Invitrogen Corporation, Carlsbad, CA, USA), according to the manufacturer's instructions. Complementary DNA (cDNA) synthesis was carried out using the PrimeScript II 1st Strand cDNA Synthesis Kit (Takara Bio Inc., Shiga, Japan), following the supplier's instruction. PCR was performed using a previously described procedure [10] with some modifications. The synthetic oligonucleotide PCR forward primer for mouse DAO was 5′‐CTGATGGCTGCTGTGAGGTA‐3′, and the reverse primer was 5′‐GGCAGCTCAGCAGGTAATCA‐3′. Primers for mouse glyceraldehyde‐3‐phosphate dehydrogenase (GAPDH) were used as previously described [30]. A total reaction volume of 20 μL was prepared containing 1 μL of cDNA sample, 0.25 μM of each PCR primer, and 10 μL of EmeraldAmp PCR Master Mix (Takara Bio Inc.). The prepared reaction mixture was run in a thermal cycler QIAamplifier 96 (Qiagen N. V., Venlo, The Netherlands). Following the enzyme denaturation step (94°C for 5 min), PCR cycles were performed with the following profile: denaturation at 94°C for 30 s, annealing at 55°C for 30 s, and extension at 72°C for 45 s. The number of cycles was set to 30 for DAO and 30 for GAPDH. After PCR completion, the reaction mixture was separated by 2% agarose gel electrophoresis and stained with a 0.5 μg/mL ethidium bromide solution.

### Western blotting and immunoprecipitation

For western blotting and immunoprecipitation analyses, mouse kidney, liver and CP tissues were homogenized in RIPA buffer (25 mm Tris/HCl, pH 7.6, 150 mm NaCl, 1% NP‐40, 1% sodium deoxycholate, and 0.1% SDS) containing Complete Mini Protease Inhibitor Cocktail (Roche Diagnostics K. K., Basel, Switzerland). Protein samples (10 μg total protein each) were subjected to electrophoresis on 5–20% polyacrylamide gels (ATTO Co. Tokyo Japan), followed by blotting onto Immobilon‐ P membranes (Millipore, Bedford, MA, USA). Membranes were immersed in EzBlock Chemi blocking buffer (ATTO Co.) and then incubated with primary antibodies (anti‐DAO antibody [10, 31], 1:100 000; and anti‐GAPDH antibody (Cell Signaling Technology, Danvers MA, USA; Cat#8884T), 1:100 000) diluted in blocking buffer. The secondary antibody used was an HRP‐conjugated anti‐rabbit IgG antibody (Cell Signaling Technology, Cat#7074P2, 1:200 000). Detection of each protein was carried out using a Chemi‐Lumi One Ultra (ATTO Co.), according to the manufacturer's instructions. Chemiluminescence signals were detected using the LAS4000 system (Cytiva, formerly GE Healthcare, Little Chalfont, UK).

For immunoprecipitation analysis, protein samples (100 μg total protein each) were mixed with 1 μg polyclonal anti‐DAO antibody. Immunoprecipitates were collected using Protein G Plus‐Agarose (Santa Cruz Biotechnology, Inc., CA, USA) and subsequently analyzed by western blotting as described above.

### Preparation of tissues for histological analyses

Mice were anesthetized with a mixture of 0.3 mg/kg medetomidine hydrochloride (Nippon Zenyaku Kogyo Co., Ltd., Fukushima, Japan), 4 mg/kg midazolam (Astellas Pharma Inc., Tokyo, Japan), and 5 mg/kg butorphanol tartrate (Meiji Seika Pharma Co., Ltd., Tokyo, Japan). Briefly, mice were perfused with phosphate‐buffered saline (PBS) using a winged needle (22G; Terumo Co., Tokyo, Japan) inserted into the left ventricle. The right atrium was cut and drained, and the blood components were thoroughly washed away. Next, the needle was stabilized by hand and connected to a gravity flow at 130 cmH_2_O, and approximately 50 mL of fixative was perfused in for about 15 min. After perfusion fixation, the brains were removed, sagittally dissected at the midline, and the segmented brains were immersed in fixative. The fixatives used were as follows: 4% paraformaldehyde (FUJIFILM Wako Pure Chemical Co., Osaka, Japan) in 0.1 M phosphate buffer (pH 7.4) (FUJIFILM Wako Pure Chemical Co.) for immunohistochemical analysis, and a mixture of 1% glutaraldehyde (TAAB Laboratories Equipment Ltd., UK) and 4% paraformaldehyde in 0.1 m phosphate buffer (pH 7.4) for immunoelectron microscopy.

### Immunofluorescence analyses

Brains were fixed overnight in 4% paraformaldehyde in 0.1 m phosphate buffer (pH 7.4). Next, 50‐μm‐thick sections were cut using a TPI Vibratome Series 1000 Sectioning System (Technical Products International Inc., Missouri, USA). The sections were then stained using a free‐floating staining method [32]. Floating tissue sections were treated with blocking buffer (PBS [Takara Bio Inc., Shiga, Japan] containing 3% bovine serum albumin [FUJIFILM Wako Pure Chemical Co.], 0.1% Triton X‐100 [FUJIFILM Wako Pure Chemical Co.], and 0.05% sodium azide [FUJIFILM Wako Pure Chemical Co.]). The primary and secondary antibodies used in this study are listed in Table 1. Nuclei were stained with 4′,6′‐diamidino‐2‐phenylindole (DAPI) (Merck KGaA, Darmstadt, Germany), and tissue sections were mounted using Vectashield (Vector Laboratories, Inc., CA, USA). Immunofluorescence tissue samples were observed using a Zeiss LSM900 inverted confocal laser scanning microscope equipped with Airyscan 2 and a Plan‐Apochromat 63×/1.4 Oil DIC M27 objective (Magnification/Numerical Aperture: 63×/1.40, Working Distance: 0.19 mm) (Carl Zeiss AG, Oberkochen, Germany). Images were acquired with the following settings: 958 × 958 pixels with pixel size 0.035 μm (33.8 × 33.8 μm regions), 16‐bit depth, and single optical section. Fluorescence from DAPI, Alexa Fluor488, and Cy3 was excited using 405 nm, 488 nm, and 561 nm lasers, respectively. Image processing and quantification were performed using Zeiss Zen 3.5 software (Carl Zeiss AG). Images were deconvoluted using the Airyscan super‐resolution processing function (Carl Zeiss AG). Following deconvolution, image analysis was conducted using Fiji (Fiji Is Just ImageJ) [33], a distribution of ImageJ [34]. Pearson's correlation coefficient and Manders' overlap coefficients were calculated using the Coloc2 plugin in Fiji (ImageJ, NIH), with automatic threshold determination according to the Costes approach [35].

**Table 1:** Antibodies used in immunostaining and immunoelectron microscopy.

Name of antibody (peptide/protein target)	Manufacturer, catalog number, and/or name of individual providing the antibody	Species raised in; monoclonal or polyclonal	Dilution used
<Primary antibody>			
DAO	Kiyoshi Fukui [24]	Rabbit, Polyclonal	1/2000
S100β	Sigma, S2532	Mouse, Monoclonal	1/2000
PMP70	Sigma, SAB4200181	Mouse, Monoclonal	1/1000
Beclin 1	Proteintech, 66 665‐1‐lg	Mouse, Monoclonal	1/1000
LAMP‐1/CD107a	R&D Systems, MAB4320	Rat, Monoclonal	1/1000
EEA1	Proteintech, 68 065‐1‐lg	Mouse, Monoclonal	1/1000
CD63	Proteinteck, 67 605‐1‐lg	Mouse, Monoclonal	1/1000
Rab11	Orinene, AB3035‐200	Goat, Polyclonal	1/1000
TGN46	Proteintech, 66 477‐1‐lg	Mouse, Monoclonal	1/1000
LC3	LMB, M152‐3	Mouse, Monoclonal	1/1000
RAB5A (E6N8S)	Cell Signaling, 46 449 T	Mouse, Monoclonal	1/1000
TSG101 [EPR7130(B)]	Abcam, ab320807	Goat, Monoclonal	1/1000
PEX5	Origene, TA501430S	Mouse, Monoclonal	1/1000
CD107b/LAMP2	Proteintech, 66 301‐1‐Ig	Mouse, Monoclonal	1/1000
GM130 (M343)	Invitrogen, MA5‐47668	Mouse, Monoclonal	1/1000
AP‐2	Abcam, ab189995	Goat, Polyclonal	1/1000
<Secondary antibody>			
Rabbit IgG	Invitrogen, A21206	Donkey, Alexa 488	1/200
Mouse IgG	Jackson ImmunoResearch Laboratories, 715‐165‐151	Donkey, Cy3	1/200
Rabbit IgG	Vector Laboratory, BA‐1000‐1.5	Goat, Biotin	1/200
Rat IgG	Invitrogen, A21209	Donkey, Alexa 594	1/200
Goat IgG	Jackson ImmunoResearch Laboratories, 705165‐147	Donkey, Cy3	1/200

### Immunoelectron microscopy studies

Briefly, mouse brains were fixed via perfusion fixation and immersion fixation in 1% glutaraldehyde and 4% paraformaldehyde in 0.1 m phosphate buffer (pH 7.4). Sections were stained using the free‐floating staining method with anti‐DAO and biotin‐conjugated anti‐rabbit IgG antibodies (Vector Laboratories Inc.). The sections were then treated with an ABC kit (Vector Laboratories Inc.) and subsequently incubated with DAB (Dojindo Laboratories, Kumamoto, Japan), followed by fixation in 1% osmium tetroxide solution (TAAB Laboratories Equipment Ltd.). The sections were embedded in Epon epoxy resin (TAAB Laboratories Equipment Ltd.). Ultrathin sections (80 nm thick) were cut and stained with uranyl acetate. The specimens were observed using a JEM‐1400 transmission electron microscope (JEOL Ltd., Tokyo, Japan) at an accelerating voltage of 80 kV.

## Conflict of interest

The authors declare that they have no conflicts of interest.

## Author contributions

KO performed the experiments; KO, TY, YS, and KF analyzed the results and created the figures; KO, TY, YS, and KF designed the research and wrote the paper.

## Supporting information


**Table S1.** Quantitative colocalization analysis of DAO and organelle‐specific markers.

## Data Availability

The data that support the findings of this study are available from the corresponding author (onok@saitama-med.ac.jp) upon reasonable request.
