# Supplementary material for: Variability in intracellular localization of D‐amino acid oxidase in choroid plexus epithelial cells
Source: FEBS J. 2026 Feb 17;293(14):4358–73. doi: 10.1111/febs.70459 (PMC13370738; doi:10.1111/febs.70459)
Supplement: Supplementary file 1 — Table S1. Quantitative colocalization analysis of DAO and organelle‐specific markers. [file FEBS-293-4358-s001.pdf]

Supplementary Table 1. Quantitative colocalization analysis of DAO and organelle-specific markers. Colocalization between DAO and organelle-specific markers was quantified using Pearson's correlation coefficient (R value) and Manders' overlap coefficients (M1, M2, tM1, tM2). Organelle markers included Golgi apparatus (GM130, TGN46, AP-2), endosomes (EEA1, Rab5a, Rab11), exosomes (CD63, TSG101), lysosomes (LAMP1, LAMP2), peroxisomes (PEX5, PMP70), and autophagosomes (Beclin1, LC3). Pearson's R values indicate the linear correlation between DAO and each marker. Manders' M1 represents the proportion of organelle marker fluorescence overlapping with DAO, while M2 represents the proportion of DAO fluorescence overlapping with organelle markers. Manders' coefficients (tM1 and tM2) were calculated using Costes' automated thresholding method to reduce background contribution. Data are presented as median ± standard deviation (SD), with the number of cells analyzed (n) specified for each marker.

Supplementary Table 1

Organella	Golgi apparatus		Endosome				Exosome		Lysosome		Peroxisome		Autophagosome	
Marker	GM130	TGN46	AP-2	EEA1	Rab5a	Rab11	CD63	TSG101	LAMP1	LAMP2	PEX5	PMP70	Beclin1	LC3
Pearson's R value	0.040±0.054	0.250±0.098	0.110±0.042	0.145±0.046	0.010±0.022	0.175±0.040	0.070±0.060	0.085±0.192	0.225±0.106	0.120±0.043	0.120±0.045	0.050±0.031	0.090±0.069	0.025±0.039
Manders' M1 (Above zero intensity of Organella)	0.015±0.007	0.525±0.092	0.361±0.186	0.315±0.226	0.002±0.010	0.751±0.052	0.154±0.100	0.266±0.260	0.134±0.068	0.314±0.137	0.450±0.093	0.062±0.295	0.090±0.160	0.019±0.013
Manders' M2 (Above zero intensity of DAO)	0.708±0.192	0.458±0.093	0.705±0.142	0.738±0.032	0.768±0.195	0.145±0.022	0.577±0.174	0.695±0.291	0.681±0.070	0.682±0.078	0.605±0.126	0.469±0.250	0.350±0.150	0.288±0.105
Manders' tM1 (Above autothreshold of organella)	0.015±0.007	0.480±0.174	0.319±0.190	0.2095±0.142	0.002±0.002	0.120±0.169	0.143±0.088	0.266±0.260	0.134±0.068	0.304±0.116	0.158±0.130	0.062±0.048	0.089±0.047	0.019±0.013
Manders' tM2 (Above autothreshold of DAO)	0.000±0.287	0.458±0.179	0.092±0.149	0.247±0.181	0.000±0.013	0.145±0.023	0.034±0.128	0.016±0.268	0.080±0.204	0.073±0.237	0.365±0.214	0.002±0.171	0.025±0.107	0.000±0.005
Number of cells (n)	17	11	11	16	14	12	16	12	20	14	18	13	15	18

Mean±SD
